# Clinical Cases in the Medical Wards of the Mercy Hospital

**Published:** 1872-12-01

**Authors:** 


### Clinical Reports.

### CLINICAL CASES IN THE MEDICAL WARDS OF THE MERCY HOSPITAL. SERVICE OF PROF. N. S. DAVIS.

REPORTED BY D. ———

CASE I. *Acute Rheumatism—Rheumatic Fever:* The case before you, gentlemen, is a middle-aged man, laborer, who was admitted into the hospital one week since, laboring under acute articular rheumatism. He had been attacked several days previous to his admission to the hospital, but what treatment he had received, if any, I do not know. You observe his expression of countenance, especially when he attempts any motion, is indicative of suffering; you find his tongue coated with a whitish fur; his pulse full, and 90 per minute; skin a little above the natural temperature, and dry; bowels rather costive; and urine scanty and high-colored.

He complains of stiffness and soreness through his back, hips, and still more in his knees, which latter have a constant gnawing pain, greatly aggravated at night. On examination, you find both knees considerably swollen and tender to the touch, warmer than the rest of the body, but not red on the surface. While the swelling is general around the joint, with no definite line of demarkation, you see a more prominent fullness on each side of the ligamentum patella, below and on each side of the attachment of the rectus femoris above, giving to the knee a characteristic oblong shape. This is produced by the effusion of serum into the cavity of the synovial membrane, which is less covered by ligaments to resist its distension at the points indicated. That the membrane is distended with serum, is readily determined by the plain fluctuation felt at the points indicated.

At the time the patient was admitted into the hospital, last week, all his symptoms were more aggravated than at present. The general fever was more active, the pains were more severe, and the articulations were swollen one-third larger than at present. The local inflammation commenced in the shoulders and spine, and had extended downward to the hips and knees, having just fully involved the last named joints at the time of admission to the hospital. This migration of the inflammation from one articulation to another, is one of the striking characteristics of rheumatism. It generally subsides in one series of articulations, at the same time that it attacks another, but not always, for we sometimes meet with cases in which it involves one part after another, until almost every joint in the body and limbs is affected, and the patient utterly helpless. Another characteristic of the disease is, that the inflamed parts seldom, if ever, suppurate. When the synovial membranes are involved, they become quickly distended with effused fluid, and the fibrous structures exterior to the membrane become filled with plastic exudation; but in neither locality is there any tendency of the effused material to degenerate into pus.

When the disease attacks the sheaths of the tendons, more especially in the wrists, ankles, and smaller joints of the hands and feet, it is accompanied by an exudation so highly plastic that it forms a firm bond of union between the tendons and the fibrous sheaths surrounding them, which, in some individuals, remains after the inflammation and swelling have subsided, causing a species of permanent anchylosis. Generally, however, the exudations accompanying acute rheumatism, are absorbed soon after the active stage of the inflammation has passed by, leaving the parts for a time somewhat stiff and tender, and for a long time predisposed to new attacks. After alluding, briefly, to the conditions of climate, and other circumstances favoring attacks of rheumatism, the Professor spoke substantially as follows, in regard to the pathology and treatment of the disease:

The fact that the general causes favoring attacks of rheumatic fever interfere mostly with the eliminative functions of the skin, in connection with the further fact that the blood and secretions appear to contain an excess of acid, and all the exudations plastic, afford strong evidence in favor of the theory which attributes the fever and local inflammations of rheumatism to an excess of some irritating acid material in the blood. It is further probable that such acid material results from the retention of effete matters, which are naturally eliminated through the cutaneous surface. Whether it is lactic acid, as claimed by Richardson, and suggested by many others, can hardly be considered fully determined. Conceding this theory of the pathology of the disease to be correct, the indications for treatment become obvious. They are, first, to neutralize the excess of acid in the system by the free use of the non-purgative alkaline salts; second, to mitigate the patient's suffering; and third, to lessen the plasticity of the exudative material in the inflamed parts, and hasten its absorption. Perhaps the best means for accomplishing the first of these objects, is to give the patient 20 grains of the carbonate, or 30 grains of the bicarbonate of potassa, dissolved in plenty of water, every two or three hours, and an occasional dose of the Rochelle salts, to move the bowels. In moderate cases, the second indication is sufficiently met by a full dose of pulv. Dover's, with a grain or two of calomel, at bed-time each night. But in the more acute and severe cases, a teaspoonful of the following prescription, given between each of the doses of alkaline salts, during the first two or three days, will aid very much, both in mitigating the pain and lessening the fever:

℞—Nitrous ether, ℥ j.

Vin. colchicum, ℥ j.

Camph. tinct. opii, ℥ jj.

Tinct. verat. viride, ʒ j.—Mix.

After the skin has become moist, the urine more free and less acid, the pulse softer and less frequent; and the inflammation has ceased to extend to new articulations, this sedative mixture may be omitted, and the patient left on the use of the alkaline salts alone, with only a dose of Dover's powder, without the calomel, at night. If, at the end of one week or more, the fever has subsided, leaving the patient pale, the skin relaxed and almost constantly bathed with perspiration, and yet the joints stiff and weak, from two to three grain doses of quinine, given three times a day, will generally be of much service.

Local applications to the inflamed articulations, are generally of but little importance; yet, in the acute stages, keeping the part constantly wrapped in cloths wet in an infusion of aconite leaves, holding in solution hydrochlorate of ammonia, will aid much to mitigate the pain.

Such is the general outline of treatment that has been found most efficacious in the treatment of acute rheumatism. When resorted to early and pursued judiciously, it will, in many cases, lead to convalescence in from seven to ten days; and there will occur a less ratio of cardiac complications than under any other system of treatment that has yet been tried.

There are some cases, however, that persist through a period of three, four, or even six weeks, despite of all treatment. There are many other articles of the materia medica that may be used instead of those already named, for fulfilling the same indications. Thus, the bitartrate of potassa may be used instead of the carbonates; or the salts of soda may be substituted for those of potassa; or the oxide of calcium, known in the shops as syrup of lime, may be used. And in cases where the colchicum purges the bowels too much, it may be replaced with double of its quantity of tincture of cimicifuga rac.

But the attention of the clinical class has been called to so many special forms of rheumatism in different parts of the body, and the corresponding variations in treatment, that it is not necessary to pursue the subject further at present.

---

The Largest Consulting Practice in the World.— Sir William Gull, of Guy's Hospital, London, is said to have the largest consulting practice of any physician in the world, worth not less than £25,000 a year ($125,000).

